# What’s so critical about Critical Neuroscience? Rethinking experiment, enacting critique

**DOI:** 10.3389/fnhum.2014.00365

**Published:** 2014-05-30

**Authors:** Des Fitzgerald, Svenja Matusall, Joshua Skewes, Andreas Roepstorff

**Affiliations:** ^1^Department of Social Science Health and Medicine, King’s College LondonLondon, UK; ^2^Interacting Minds Centre, Aarhus University, Aarhus CAarhus, Denmark; ^3^Department of Culture and Society - Section for Anthropology and Ethnography, Aarhus UniversityHøjbjerg, Denmark

**Keywords:** Critical Neuroscience, experiment, critique, the social, sociology of neuroscience, optimally interacting minds, interdisciplinarity

## Abstract

In the midst of on-going hype about the power and potency of the new brain sciences, scholars within “Critical Neuroscience” have called for a more nuanced and sceptical neuroscientific knowledge-practice. Drawing especially on the Frankfurt School, they urge neuroscientists towards a more *critical* approach—one that re-inscribes the objects and practices of neuroscientific knowledge within webs of social, cultural, historical and political-economic contingency. This paper is an attempt to open up the black-box of “critique” within Critical Neuroscience itself. Specifically, we argue that limiting enactments of critique to the invocation of context misses the force of what a highly-stylized and tightly-bound neuroscientific experiment can actually do. We show that, within the neuroscientific experiment itself, the world-excluding and context-denying “rules of the game” may *also* enact critique, in novel and surprising forms, while remaining formally independent of the workings of society, and culture, and history. To demonstrate this possibility, we analyze the Optimally Interacting Minds (OIM) paradigm, a neuroscientific experiment that used classical psychophysical methods to show that, in some situations, people worked better as a collective, and not as individuals—a claim that works precisely against reactionary tendencies that prioritize individual over collective agency, but that was generated and legitimized entirely *within* the formal, context-denying conventions of neuroscientific experimentation. At the heart of this paper is a claim that it was precisely the rigors and rules of the experimental game that allowed these scientists to enact some surprisingly critical, and even radical, gestures. We conclude by suggesting that, in the midst of large-scale neuroscientific initiatives, it may be “experiment”, and not “context”, that forms the meeting-ground between neuro-biological and socio-political research practices.

## Introduction

What is there still to say about the growth and prominence of the new brain sciences? Almost 10 years ago, Steven Rose (2005) pointed out that “the global scale of research effort now put into the neurosciences primarily in the US, but closely followed by Europe and Japan, has turned them from classical ‘little sciences’, into a major industry engaging large teams of researchers, involving billions of dollars from government…and the pharmaceutical industry” (Rose, 2005: 3). With the recent advent of the Human Brain Project (Honigsbaum, 2013), and the BRAIN initiative (Markoff and Gorman, 2013), this narrative of growth and expansion has scarcely changed. Certainly, if their influence (and desire for influence) is sometimes over-stated, neuroscientific spaces are now among the most potent and creative sites for understanding human beings, their subjectivities, and their societies (Andreasen, 2001; Pickersgill et al., 2011; Rose and Abi-Rached, 2013).

Unsurprisingly, as the neurosciences have grown in size, prominence and prestige, so has critical sociological, philosophical, and historical analysis grown up around their foothills (Martin, 2000; Dumit, 2004; Ortega and Vidal, 2007). Such works range from critiques of a frightening “neuro-reductionism” (Martin, 2004), to interest in the philosophical implications of neuroscience (Malabou, 2008), to a more delicate labor of carving out shared spaces of interest between the neuro-and social sciences (Roepstorff and Frith, 2012; Rose and Abi-Rached, 2013; see also Fitzgerald and Callard, Forthcoming, for an analysis of these modes of engagement). In recent years, however, “Critical Neuroscience” has emerged as perhaps the most prominent, and certainly the most self-consciously “critical”, framework for thinking about the relationship between neuroscience, society, politics and economics (Choudhury et al., 2009; Slaby, 2010; Slaby and Choudhury, 2012). Put crudely, scholars within this tradition, sometimes rooted in the Frankfurt School, and usually tilting at the hidden political and economic entanglements of neuroscientific assumptions, try to pull the experimental rhetoric and practice of neuroscience *away* from an organizing fantasy of distanced facthood, and *towards* a more concretely political and reflexive socio-critique –re-inscribing the objects of neuroscientific practice back *within* the webs of social, cultural and historical context to which they are always inevitably subject (Choudhury et al., 2009).

What follows here is also an essay on neuroscience and critique. The paper is not a tediously scholastic disagreement with Critical Neuroscience: we have found too much of value in its corpus, have learned too much from the scholars within it, and have shared in too many events that have explored and expanded its core rubrics. Moreover, as scholars who labor in, on, and through, the contemporary neurosciences, we remain sensitive to the acuity of, and creative potential in, a critical insistence on the looping relationships between facts, politics, ideologies, and publics. But we also think that there are important *lacunae* in the growth of Critical Neuroscience. In this paper, we re-visit the relationship between neuroscience and critique, to propose that a more “critical” neuroscience is not only one that is attentive to its own context; sometimes, we suggest, a critical neuroscientific statement is produced through precisely the opposite strategy—by focusing on, and working *with*, the internal, world-excluding dynamics of the neuroscientific experiment. Our core argument is that limiting enactments of critique to social or historical context misses the force of what a highly-stylized and tightly-bounded neuroscientific experiment can actually *do*. We will show how the neuroscientific experiment may *also* enact critique, in novel and surprising ways, formally independent of an attention to the workings of society, or culture, or history.

We center this argument on one case study: a series of experiments called “Optimally Interacting Minds” (OIM), published in *Science* by Bahador Bahrami et al. in 2010. Two things interest us about this study: (1) it is made-up of a tightly-bound series of experimental demonstrations, cleaving closely to the conventional rules that make up the experimental game of psychophysics; and (2) it enacts and legitimizes a number of potentially “critical” interventions about the virtues of social and collective life, about the suboptimal performance and reasoning of the private individual, and about the nuanced—and deeply political—relationship of evidence and knowledge to forms of shared decision-making. We use this experiment to argue that “experiment”, as much as “context”, is a good basis for bringing a more “critical” neuroscience into being. But we will also suggest that such a focus may require the dilution of a well-worn (and perhaps, now, rather comfortable) insistence that the neuroscientist needs to focus on the indelible presence of “society” and “history” and “politics” and “economics” within her procedures. It requires commentators to think also about the rules of the experimental game itself, about the procedures that grant it its potency, and about the kinds of statements that those procedures make possible.

The paper is in four parts: first, drawing especially on three programmatic texts (Choudhury et al., 2009; Slaby, 2010; Slaby and Choudhury, 2012) we offer a short exegesis of Critical Neuroscience, isolating what, precisely, is intended by the word “critical” within it; we then offer a brief account of the boundaries of experimentalism in social psychology and neuroscience; third, we introduce the OIM experiment, locating its core critical intervention in the broader sweep of neuroscientific experimentation; in the final section, we argue for a new way of relating to the neuroscientific experiment, whose rules and traditions, arcane as they seem, are not so isolated from critical and political statements as they sometimes appear. We conclude with a suggestion that, *pace* much discussion hitherto, it might be “experiment”, and not “context”, that forms the critical meeting-ground between neuro-biological and socio-political research practices, in the 21st century.

## Locating critique

At its heart, Critical Neuroscience is an attempt “to respond, both philosophically and scientifically, to the impressive and at times troublesome surge of the neurosciences” (Slaby and Choudhury, 2012: 29). Authors within this genre are not working to destabilize neuroscience: their more parsimonious and constructive goal is to question the broader cultural urge towards neuroscientific explanations, to point to the problematic bases both of this urge *and* of the brain science it wills into existence, and to imagine, beyond both, a different sort of neuroscience—one that is able to question its own “givens” and to recognize its own history and context; a discipline in which “historical, anthropological, philosophical, and sociological analysis can feed back and provide creative potential for experimental research in the laboratory” (ibid.: 29–30). At the center of this enterprise is a single qualifier: “critical”. It is the will to *critique* that legitimizes this programme, that drives it forward, and that organizes resources around it: “grounded in a framework of critical theorizing”, Choudhury et al. (2009) write elsewhere, “and in view of the social and cultural factors that shape research agendas and theories, Critical Neuroscience suggests ways to equip neuroscientific research with basic tools of critical practice” (Choudhury et al., 2009: 74).

But what, exactly, is the “critical” in “Critical Neuroscience”? Confronted with what they see –following Axel Honneth—as an emerging set of “social pathologies of reason” between the neurosciences and their contexts, Critical Neuroscience scholars interpret their task as opening up the black box of scientific facthood, and unveiling the deeply contingent socio-historical logics embedded within the neuroscientific fact (ibid.: 65 *cf*. Martínez Mateo et al., 2013). But what we wish to briefly explore here is the intellectual history and disciplinary genealogy mobilized by this term—including the forms of political, epistemological and economic commitment that are both held together *within* it, and excluded *from* it. If there is not space in this paper for an intellectual history of the urge to “critique” as such (see de Boer and Sonderegger, 2012), still we want to situate the use of this term more precisely within the broader *oeuvre* of a Critical Neuroscience.

Within that literature, various intellectual forebears are claimed for critique, including Kant (Slaby and Choudhury, 2012), Axel Honneth (Kirmayer, 2012), and even Bruno Latour (Slaby, 2010). But if it seems difficult to assemble this inheritance into a coherent programme, in practice its articulation leads to a number of specific looping interests. For Choudhury et al. (2009), a “critical” approach to neuroscience is an unmasking of the scientific “brain-fact”, a conceptual and anthropological exposure of the journey that mental phenomena take on their way to facthood, and an undermining of the political and economic contexts, media interests, lay perceptions, and so on, that are braided through that “fact” along the way (Choudhury et al., 2009: 65). For Campbell (2010), a year later, a critical neuroscience is straightforwardly positioned as one “more attuned to the ‘social”’, (Campbell, 2010: 101). “What is at stake”, Campbell argues, is precisely “how social factors will be addressed—who is best positioned to address them through what vocabularies and with what goals?” (ibid.). For Kirmayer (2012), a critical approach directs attentions specifically to the forms of cultural reasoning underpinned by the “neuro” prefix: critique, in Kirmayer’s account, “is our vehicle through which to focus on popular culture, as well as neuroscientific and technical rationality and their economic and political motivations” (Kirmayer, 2012: 367).

What binds these understandings together, and legitimizes the use of “critique” within Critical Neuroscience, is a commitment to a particular form of *politics—*a politics that is otherwise taken to be effaced from the rhetorical and experimental game that entangles the neurobiological mainstream. This commitment is perhaps most clearly expressed in Slaby and Choudhury’s (2012) “Proposal for a Critical Neuroscience” (2012) where they argue that that their venture “opens up a space for inquiry that is itself inherently and self-consciously political” (29). For Critical Neuroscience in general, this space is rooted in “the persuasion that scientific inquiry into human reality tends to mobilize specific values and often works in the service of interests that can easily shape construals of nature and naturalness” (ibid.). Or as they put it elsewhere in the same text, the overarching goal is to “analyze the allure and functions of the *neuro* in the broader scheme of intellectual and political contexts” (ibid.: 45). And again: “Critical Neuroscience should not stop at description and complexification”, being concerned instead with the “depoliticalization of scholarship in the face of the increasing commercialization of academia…a more radical and openly political positioning is needed in [the] face of these trends” (ibid.: 31).

Here, we draw an important distinction: for Critical Neuroscience, the argument is not that an *a*political scientific practice should suddenly *become* political; there is no assumption at all that there is no politics in neuroscience as it is currently constituted. Instead the argument is that: (1) a falsely *de*politicized rhetorical and conceptual apparatus of neuroscientific experimentation and dissemination has excised, or hidden, the inherent political inputs of the neuroscientific enterprise, in its pursuit of a neutral-looking facthood; (2) said apparatus would be better served if it began to recognize the political assumptions and priorities that are always-already *in* it; and (3) in particular, the same apparatus should distance itself from a misguided rhetorical game of distanced facthood, which merely draws a veil over, and thus reifies, the cultural and political biases that are always (inevitably) in the experiment. It is thus not neuroscience as such, but the (broadly understood) material-semiotic *experimental game* of neuroscientific facthood, and what the game is understood to be *for—*including its manifestations in hypothesizing, disseminating, translating and so on—that is at stake in this claim for a re-politicization of the neuroscientific enterprise.

The immediate target and direction of a re-politicized neuroscientific apparatus, and of the neurobiological fact it draws forth, takes different forms. In the “Proposal”, particular emphasis is placed on “the commercialization of research…[such that] sociological analysis can highlight the pressures that commercial, pharmaceutical, and military interests place on neuroscience”—especially given that “scientists are not usually trained to be very sensitive to the subtleties of, and social conflicts within, political and institutional environments” (ibid.: 43, 39). Elsewhere, the authors seek

a discursive space for debate both in professional and practical domains about the categories and application of neuroscience, and about related social issues such as the organization of labor, conception of health and disease, goals and practices in parenting and education, issues about law and punishment, technological self-optimization, and much more (ibid.: 40).

In other texts, authors point to the burgeoning relations between neuroscience and national security industries (Marks, 2010); the growth of a pharmaceuticalised biological psychiatry (Kirmayer, 2012); the removal of “the social” from conceptions of addiction (Campbell, 2010); the troubling relationship between neuroscientific findings and management techniques (Slaby, 2010); the neurobiologization of crime (Choudhury et al., 2009)—and so on. In each case, whatever the locus of attention, the call to “critique” is a call to make manifest, and then reform, the political *in* the neuroscientific experiment, to which extent politics is located in moments and processes where such experiments, and the rhetoric of facthood that surrounds them, have an effect on, or are taken up by, actors with commercial, governmental or other neoliberal ends, thus having reactionary outcomes within the mundane politics of crime, labor, illness, security, and justice.

That such issues look like the bread and butter of a traditionally-minded social science is not a coincidence. For it is “the social”, understood in its unreconstructed Durkheimian sense, that gathers up and justifies this remit: “critical neuroscience puts particular emphasis on the social” argue Slaby and Choudhury (2012): 36. Against (what they see as) a dominant “actor network theory” approach within the study of scientific practices and experiments, Slaby and Choudhury insist on “the social” as “a potential explanatory resource”—allowing analysts to avoid a quietist “neutral” cartography, and instead to “penetrate beneath the surface of emerging practices, relations, and styles into the dynamics of power that may shape or stabilize surface phenomena, facilitate or hinder certain alliances or actions” (ibid.: 37; cf. Rose, 1996; Durkheim, 1982 [1895]). These authors, of course, are perfectly aware that, in the early 21st century, such an approach is somewhat *passé*: they explicitly refuse an old-fashioned account of the social as a view-from-nowhere *explanans* of all human phenomena, and stress (indeed, precisely following the actor-network approach that they are elsewhere suspicious of; see Callon, 1986) the processual “assemblage” of this “social” within a multitude of actors and practices. But thus trying to find a way—not always clearly or convincingly—between very different ways of conjuring the social, they refuse quietist tendencies embedded in recent sociological attention to scientific practices, insisting that “the activity of assemblage, in our sense of the term is …an inherently *political* one” (Slaby and Choudhury, 2012: 38, my emphasis).

This reliance on a self-consciously “political” socio-critique in the old style is, of course, perfectly respectable—if not very fashionable. Still, an important tension comes into view here: if these authors are committed to an epistemological politics suspended somewhere between Kant and the Frankfurt School, they are aware that this way of understanding critique, and this mode of doing politics, have both been convincingly superseded within the very social science literature upon whose methods and perspectives they are so reliant (e.g., Latour, 2004). What is centrally at stake for Critical Neuroscience, then, is an attempt to enact a form of critical attention to the neurosciences, from the point of view of the social—at a moment in which *precisely* such a mode of attention has been orphaned by the intellectual practices who claim the social as their own, and who thus form the empirical and theoretical ground upon which Critical Neuroscience seeks its contribution. Perhaps the fullest expression of this tension comes in an article by Slaby (2010). “The challenge”, he writes, “is to render ‘critique’ meaningful again in a time when this notion has fallen into disrepute in mainstream thought and theory” (Slaby, 2010: 410). For Slaby, Latour’s re-invigoration of scientific facthood as “matters of concern” (i.e., stable entities whose stability is nonetheless an ongoing achievement; see Latour, 2008) permits (*contra* Latour’s own view) a re-invigoration of the role of critique—which, for Slaby (2010), is not a mere debunking of scientific authority, but a much more generative and constructive enrichment of scientific facthood, now thickly embroiled with matters of “human interest” (Slaby, 2010: 411). Critique, in Slaby’s account, calls political attention, amid the neutral-looking assembly of matters-of-concern, to who or what is doing all that concerning—and to whose concern gets excluded at the same time. For Slaby, “the assembling [of] matters of concern from multiple perspectives can provide a balancing force against the monopoly of experts and specialist associations” (ibid.). Here, “context” is the lever: the task is “to reinscribe the relevant influences and multiple causal factors, point to historical trajectories, and record cultural understandings and differences” (ibid). The summoning-up of context does not efface the neurobiology of a topic like addiction, but instead provides a “much richer” account that can build upon the “meager construal” of a neuronal reductionism (ibid). The work of the critic, then, is to identify interests, to seek out pathologies of reason, to begin a transdisciplinary discussion about the normative underpinnings of these interventions—and then to include all of this in some specific neuroscientific practice or encounter (ibid.: 411–412).

Given that Latour has been invoked by these authors themselves, we may rely on his rubric to point to some of the problems here. Certainly, authors within Critical Neuroscience want critique not to be the debunking, denouncing, killjoy of yore, and they situate the critical urge as a positive, additive function in the practice of neuroscience. But they remain committed to a view of, specifically, a neuroscientific *research-practice* which is: (1) de-politicized through its firm rhetorical exclusion of human social and cultural worlds (of interests, ideologies, economies, and so on); but also (2) nonetheless irretrievably political, if only in the sense that, by drawing a veil across these interests and ideologies, it reifies the (troubling) contexts in which its own performance takes place. At the heart of this view, then, is an idea that the experimental game of neuroscience *itself* relies on a distinction between, on the one hand, things that are human, and political, and economic, and unavoidably inflected by context, and, on the other hand, things that are scientific, and biological, and embodied, and best understood in laboratories. To put it another way, the argument of Critical Neuroscience is that even if the neurobiological sciences remain, at base, deeply human, cultural and political endeavors, the atomized brain-fact, and the experimental game that produces that fact, are the products of a technified attempt to exclude, cover-over, or simply ignore that human, cultural and political base. A better—more critical—research-practice is one that calls a halt to this misguided game of exclusion, denial, veiling, and so on.

If the committed Latourian surely agrees with Critical Neuroscience that some kind of politics, or interest, or agency, is inseparable from the generation of brain-facts, we will hardly find room in her thought for the imagination of a research-practice which succeeds *precisely* by relying on a distinction between the human world of context and politics, and the laboratory arena of neutrality and facthood. For Latour, of course, and quite unlike the core claims of Critical Neuroscience, there is no successful or sustainable fact-producing machine, no meaningful theatre of experimentation, to which politics and interest have been rendered external; there is no God-given task for the philosopher or the sociologist to “piece together the social, political, economic, social and cultural factors involved in the development of neuroscientific insights” (Choudhury et al., 2009: 65). If Critical Neuroscience seeks a space for its own analysis in the “the selective attitude and methodological reductionism of experimental approaches” (Slaby and Choudhury, 2012: 25), Latour reminds us that there is no experimentally-generated fact, no disseminated finding, no “depoliticalized” “matter of concern”, which has not always-already been generated, sustained and circulated precisely through a careful attention to, and cultivation of, its own complex bundle of interest, conviction, care, deviousness, misunderstanding, hope, hucksterism, and so on—*the careful assemblage of which is the sole guarantee of successful “facthood” in the first place*. Put more simply: there is, in Latour’s account, no successful scientific object, and no potent experimental practice, to which interest, context, politics, and democracy have been successfully rendered external, except in the most trivial sense. There can be no additive function for “critique”, moreover, *unless* it is founded on the idea that successful experimental practices are about producing facts without, or exclusive of, or even only in denial of, context, and discussion, and politics, and interest—an assumption, in other words, that we would have better facts, and more democratic ones too, if these externalized relations were much more on the surface, much more reflected-upon, much more open to *critique*. This is, finally, how we must understand the critical gesture at the heart of Critical Neuroscience. Despite appeals otherwise, it is locked within a social-scientific and philosophical literature whose organizing premise is that elaboration of the political is *external* to the rules of the neurobiological-experimental game, at least as it currently stands. That game is what we turn to in the next section of the paper.

## Re-thinking the experimental

Of course, experimentation has been a central object of investigation in histories and philosophies of science for some time—and we cannot do justice to a long literature here (Kuhn, 1976; Bachelard, 1984 [1933]; Rheinberger, 2001). In recent decades, however, Hans-Jörg Rheinberger has brought discussion of experimental maneuvers to the forefront of histories of science, stressing that research does not typically begin with the choice of a theoretical framework, but in fact with the choice of a specific technological system; for Rheinberger (2001), it is the experimental system, and not necessarily the hypothesis or the theory, that lies at the center of the knowledge-production process (Rheinberger, 2001: 19, 21f.). For the psychological sciences (including social psychology) especially, with their wavering affiliations to “science” as such (Ash, 1992), there are well-known accounts of these disciplines’ historical trajectory *towards* experimentalism, and into positive science (Danziger, 1992; Greenwood, 1994). Danziger has stressed, nonetheless, that this trajectory is not self-evident—since none of the emerging social sciences were experimental in their methodology (Danziger, 2000: 331). The emergence of an experimental rubric for (social) psychology thus required the imagination of a specific—and decidedly non-neutral—relationship between method and object: for pioneering social psychologists like Floyd Allport, Danziger points out, “it was impossible to advance a credible case for a methodology of scientific experimentation on any social object without redefining that object in a nontraditional way” (ibid.: 332–3.).

Thus, early experimental psychology employed methods that were “limited to exploring effects that were local, proximal, short term, and decomposable” (ibid.: 334). These conventions were made possible, of course, by redefining prior notions of the social in social science, which in turn were based on “effects that were non-local, distal, long-term, and experimentally non-decomposable” (ibid.). As empirical investigation in social psychology developed, and in common with developments elsewhere in the psychological sciences, statistical methods moved to the forefront of experimental technique (Porter, 1996). It was then a short step for statistical significance to become the experimental apparatus of choice “for decisions about the validity of psychological hypotheses” (Danziger, 1994: 154). Again, we skate over a great deal of complexity here—but what interests us is the role of this complex “surface of emergence” within social-psychological experimentation, especially as that rubric has more recently become entangled in the neurosciences. Cromby (2007), for example, reminds us that experimenting with social life in the neurosciences is a process of generating fixity over contingency, of emphasizing social cause over social influence, of replacing collective representation with embodied reification, and so on: the risk for experimenters, Cromby points out, is in confusing “rigidly measured differences between experimenter-constituted groups obtained in highly artificial circumstances” with “actual social processes in everyday life” (Cromby, 2007: 164). As Simon Cohn (2008) shows, in social neuroscience experiments especially, the “social” gets mapped onto the brain and has to be conceptualized as a material object: the experimental focus is not on the space of interaction, at what people do or what happens between them; the interest is directed towards what happens within the individual brain (Cohn, 2008: 100).

These descriptions, and cautions, about the emergence of an experimental game within social psychology and neuroscience, and especially about how the rules of that game figure social life, are well taken. And yet what interests us is less the historicization or contextualization of that game as such—and more what its conventions and its rules allow us to do, and to say. At the risk of appearing naïve, in what follows we will temporarily set aside our usual suspicions and ironies about this game, and about the broader forces and relations of power that impinge upon it. If experimental facthood in neuroscience—even the idea of such facthood—is a function of context-dependent rules, then what follows here is an optimistic story about the *affordance* of context, and not another lamentation about its *constraint*. We are fundamentally interested, first, in what a highly-stylized and tightly-bounded neuroscientific experiment can actually do, and, second, in the relationship between the limits and rules of this game, and the enactment of specific kinds of statements. The term “statements” is deliberately vague, here—we use it to draw attention to the broad enunciative lifeworld of neuroscientific experiments, and to the kinds of things that particular experiments make it possible to think, and to say. Consider, in this sense, a seminal study in social neuroscience—Tania Singer et al.’s studies on pain and empathy, published in *Science* in 2004 (Singer et al., 2004). Singer investigated how women, lying in an fMRI scanner, responded neurally to pain experienced by their romantic partners. The paper begins with a general observation: “Human survival depends on the ability to function effectively within a social context” (Singer et al., 2004: 1157). The authors emphasize that effectively functioning in such a context is embedded in the capacity to understand “others intentions and beliefs”—but it also requires empathy, or “being able to understand what others feel” (ibid.). The experimenters thus take 16 couples, placing one member of each in the scanner, and the other elsewhere in the room. Both are given a pain stimulus, and fMRI measures are taken from the subject in the scanner *both* during the reception of her own stimulus, and, separately, while she sees her partner receiving a pain stimulus. To cut a complex story short: similar brain regions were active both when the subject in the scanner received the stimulus *and* when she was given a signal that her partner was receiving the stimulus (recruiting brain regions associated with the affective dimensions of pain, but not the sensory dimensions).

In one sense of course, and without wishing in the slightest to denigrate this fascinating study, one might say that—aside from neuroanatomical specificity—there is a certain *mundaneness* to its conclusion. Who after all, is surprised to learn that emotions are contagious, or that we feel the pain of others, or that if you are romantically involved with someone, you probably know how that person feels in certain situations? But, as the experimental situation unfolds, a kind of *strangeness* emerges too. Because we begin with a very artificial-looking, densely-rule-bound situation: in the experimental setting, a person lying in the scanner must rate the level of pain of her loved one, whose hand she only sees on a tilted mirror. And she knows that this “pain” is only a small, controlled prick in an experimental setting; there is no major trauma. And yet, harnessing the force of its measures, this rather artificial construct of experimental rules and regulations, as applied to the empathic experience of pain, is, nonetheless, skillfully—and convincingly—traced to a very general, and very striking remark about the survival of the human species in general, and the role that social context and empathy plays in that survival—via a rather specific and carefully demonstrated observation that understanding the feelings of others has a comparable neural architecture to understanding our own feelings.

It is this dynamic of the strange and the mundane that we wish to keep in play here, and especially in our discussion of the experiment that follows. Because, in both of these studies, what appear to be highly artificial claims about very commonsensical phenomena, can nonetheless enact, legitimize, and authorize, some very striking, and potent, and not at all obvious, claims about the social world and its human inhabitants in general. The temptation for the external observer is to only focus on one half of this dynamic: but if we focus only on the *strange* half (on the tightly-bound, carefully-quantified artificial game that allows us to say something convincing about empathy, and about human social life in general), then we risk taking the whole thing rather too seriously: it is a powerful biological reductionism making very grand claims; it quantifies and neurobiologizes human social life; it locates evolutionary history in romantic partnership, and so on. On the other hand, if we only focus on the *mundane* aspects of the experiment, we run the opposite risk—we do not take it seriously enough, insisting that all it does, really, is reproduce, in a highly complex technocratic language, in neatly quantified and modeled form, something that of course we have already known for many years. What we want to do, in our consideration of the experiment that follows, is hold onto both halves of this binary, and focus on the dynamic *between* them. What would happen if we took the experiment just seriously enough? Could we learn to see it as a game, with particular rules, and constructs, and rituals, that—played well—sometimes allows the generation, and then also the legitimation, of some very remarkable statements? Could we see it as a game in which the rules and conventions might be manipulated, and played-around-with, such that *other* kinds of statements might become possible? Most importantly: could a *critical* understanding of an experiment be one that seeks to understand, to replicate, and even to admire, the style of an experimental game that makes a particular statement possible? Might we even, pursuing this inversion, describe a *naïve* analysis of an experiment as one that takes its own sudden awareness of the game to be both the start- and end-point of considered investigation?

By a game “played well”, here, we refer only to an experimental demonstration made convincingly and sustainably—i.e., one that has achieved force, or gained strength, to draw again on the Latourian vocabulary (Latour, 1988: 158–162). Our interest is in the relationship between such demonstrations and the possibility of subsequent “statements”—statements, for example, as above, about the entanglement of social relationships, affect, and human survival; or, indeed, as we will discuss below, about the connections between sociality, co-operation, communication, and success. We stress that we are not trying to construct a timeless or universal rubric for the analysis of experiments—questions about power, about the implications of experiments, about historical context and so on, surely remain pertinent. What we add here, much more modestly, is just *another* way of thinking about neuroscientific experiments—one that is not currently prominent within the critical literature, and yet that might (this is the core gambit of the paper) have the capacity to significantly diffract how we imagine the experiment’s relationship to critique, and to politics.

## An optimally critical experiment

The OIM experiment was conducted in Aarhus (Denmark) by Bahador Bahrami and a group of researchers affiliated with Aarhus University and University College London (Bahrami et al., 2010). The purpose of the experiment was to determine the information-sharing conditions under which a pair of individuals, co-operating to make perceptual judgements about abstract visual stimuli, might outperform the judgements of the best individual in that pair; the authors were interested in “how signals from the same sensory modality (vision) in the brains of two different individuals, could be combined in a social interaction” (1081). Stated more plainly, the purpose of the experiment was—as the abstract suggested—to test the truth of a well-known cliché: “are two heads really better than one?” (ibid.).

In this section, we use this experiment to illuminate the ways in which dynamics between the strange and the mundane can be enlivened within the experimental space of a contemporary neuroscience. But we also dwell on this experiment because it helps to show, perhaps under the bland surface of much social psychology and social neuroscience, the potential for some unexpected traffic between enactments of “the social” and rhetorics of “critique”—a traffic that, we claim, is occluded by the insistence on a rigid separation between these domains. In some ways, the question posed by the OIM experiment could scarcely have been more humdrum. At the same time, the experiment also significantly rippled the epistemological surface from which it grew—such that it has now come to play an important critical role in relation *to* that background, and in particular to the way that it has both imagined and enacted specific iterations of “the social”. There is hardly space here to give an adequate account of the histories of social and behavioral psychology (for which see e.g., Danziger, 1992; Greenwood, 1994)—nor, indeed, can we do justice to the wavering history of “the social” within the economic, behavioral, psychological, and social sciences (Rose, 1996), or to the often subterranean critical psychologies that have quietly torqued this history (Burman, 1994). Our goal is slightly different: in pursuit of some critical imperatives that are already *in* contemporary neuropsychology experiments, we take the OIM experiment as exemplary of a specific field of possibility. This will require some unavoidably broad historical brushstrokes, in order to give a sense of the wider surface of emergence.

Among the foundational themes of 20th century, Anglo-American empirical social psychology was a concern with how peoples’ judgements and behaviors were influenced though interactions with others (Allport, 1920; see Parkovnick, 2000, for a contextualization of this programme). If the “social” in “social psychology” has not always been clear (Greenwood, 1994), and indeed, social psychology itself an often disunited science of differentially “social” traditions (Good, 2000)—nonetheless, we might broadly see, within the advent of a “social” psychology, the assemblage of experimental and conceptual spaces for imagining forms of motive and intent *beyond* the individual. But this relationship has not always made for happy inferences. Solomon Asch’s (1952, 1956) experiments on group pressure and conformity are perhaps some of the best-known exemplars of this phenomenon: Asch presented groups of participants with cards showing a reference line alongside a set of comparisons, and asked members of the group to report which of the comparison lines was equal in length to the reference. Of course, as is now well known, each group contained only one real participant; the rest of the group was made up of experimental confederates, instructed to give obviously false judgements on a subset of pre-ordained trials. Asch found that participants often went along with the group consensus, even though it was obviously wrong, even though the confederates were all strangers, and even though there were no incentives to conform and no penalties for defecting. What was particularly striking about Asch’s experimental results is their suggestion that subsuming one’s own judgement to that of the group might not be strategic; that it might have no moral or material end—that it might be something much more banal, a kind of path of least resistance in the face of social influence.

As a metonym for social psychology, Stanley Milgram’s (1963, 1965) obedience experiments perhaps loom even larger in the popular imagination than Asch’s work. In Milgram’s studies, people were told that they were participating in an experiment on learning, and that they were to complete the task with another person. Again, as is now well known, each participant was told that she was assigned the role of “teacher”, while the other participant—who was in fact an actor—was assigned the role of “learner”. The experiment consisted of a series of trials in which the teacher was required to quiz the learner: for every mistake the learner made, the teacher was to administer (what they thought was) an increasingly powerful electric shock. The finding that has become most centrally associated with these experiments is that a majority of people were willing to administer the electric shocks, beyond an apparently safe level, when asked to do so by an authoritative other. What interests us, however, is the way in which Milgram’s studies extended the normative scope of the statements produced by Asch’s: people did not merely conform to social influence; they did so even to the point of causing serious harm to others. “The social psychology of this century”, Milgram (1974) himself would later reflect, “reveals a major lesson: often it is not so much the kind of person a man [sic.] is as the kind of situation in which he finds himself that determines how he will act” (Milgram, 1974: 205).

Clearly, there is much to be said, here, about the mid-century and post-war historical context of these studies, and their intense focus on “intergroup relations, leadership, propaganda, organizations, political (e.g., voting) behavior, economic (e.g., consumer) behavior, and environmental psychology” (Pepitone, 1981: 977). Within such a space, it is not hard to understand how scholars may have focused heavily on the susceptibility of people to the form of persuasive messages over content (Tversky and Kahneman, 1981); the fragility of the links between people’s convictions and their actions (Darley and Batson, 1973); the impossibility of individual, conscious free will (Wegner, 2002), and so on. At stake in all of these experiments are two core claims: (1) that some motive force outside of, or beyond, the individual, might drive particular instances of actions and belief; and (2) that when people form a group to interpret uncertain information, reach decisions, and plan for action, the outcome is often not good. We situate these interests not only in a post-war concern with propaganda and inter-group relations, but also in wider social and economic trends, manifested not least in the psychological sciences, that, at least for much of this period, elevated the individual over the collective: as Danziger (1994) again reminds us, throughout the 20th century, much institutionalized, mainstream psychological research has gradually found itself in agreement with the late-capitalist notion of an “independent individual for whose encapsulated qualities all social relations are [or should be] external” (Danziger, 1994: 296).

Over time, of course, this marginalization of the positive qualities of social interaction—what has been called the “negative bias” in social psychology (Sheldon and King, 2001)—has been drawn into question (It is worth nothing that this bias does not exist everywhere: different views on the virtues of collectivity and teamwork have long persisted especially in the organizational and management literatures. See Guzzo and Shea, 1992, for an overview). This “negative bias” is precisely the context in which we wish to understand the OIM experiment, which tested a cliché—that two heads are better than one—that was nonetheless at odds with that tendency. Pairs of participants were asked to look at two sets of visual stimuli, presented in sequence. In one set, the contrast of one of the elements was slightly higher. The task was to identify which set—the first or the second—contained the stimulus with the higher contrast; participants first performed the task alone, after which they were forced to make a joint decision. The rules of the experimental game allowed for four models of information sharing: (1) When participants disagreed in their judgements, only the isolated individual judgements were shared, and the joint decision was decided randomly (coin-flip or CF model); (2) Individual judgements were again shared, but the pair learned (from feedback) which person had the better track record, and weighted their joint decision accordingly (behavior and feedback or BF model); (3) Participants shared not only their individual decisions, but also their degree of *confidence* in their decisions, and weighed up their joint decision (weighted confidence sharing or WCS model); and (4) Participants directly shared the parameters of their sensory representations, as if these representations were somehow transmittable through a direct neural channel (direct signal sharing or DSS model).

Without wishing to go into excessive detail—the four models were fully specified quantitatively, and, within the contrived experimental context, made distinct quantitative predictions, which could straightforwardly be distinguished. With all trials completed, the experimental data ultimately supported the WCS model, showing that although people cannot communicate their perceptions directly, they *can* and *do* improve their performance by comparing their confidence *about* those perceptions. The implications of this finding were striking: not only could people effectively communicate the certainty of their judgements, *this ability allowed them to outperform the best member of the pair* (The only catch was that people’s individual perceptual sensitivities should not be too different from one another, and they should be fairly accurate in evaluating how confident they were about their own judgements). So contrary to the negative view of social interaction prevailing in empirical social psychology at the time, two heads really can be better than one. The qualities of the “independent individual” have perhaps been radically over-stated.

Of course, this is only one experiment—a single case study—and on its own it hardly moves the entire field of social cognitive neuroscience towards a more expansive view of “the social” as such (at least in the view of commentators who find the neurosciences reductionistic on this score). Nor does it suffice to demonstrate the over-arching point we’re gesturing at. Indeed, many will point out that even here, irrespective of the result, what is at stake is still some kind of “biologization” of the social—and that *that* (including all the imperatives that are contained within it) should be the focus of critical attention. Others will argue that OIM is only another kind of falsification—that it leaves the broader socio-political contours of the discipline untroubled. But, as we argued at the beginning, what interests us here is neither a dramatic revision of Critical Neuroscience nor some major event in social psychology and neuroscience. Our goal is a more modest one: we only want to suggest that, looked at in particular sort of way, and interpreted in terms of a specific history, OIM might be indicative of a more nuanced relationship between critique and experiment than has yet been allowed in these literatures. We do *not* say that there is anything about experimental design—still less the specific design of this experiment, which may well have produced a different kind of finding—that guarantees a “progressive” or a “critical” result (nor, of course, was there intended to be). What we are ultimately trying to show is only that the rules of the neuroscientific and psychological experimental game—and in particular that game’s insistence on associating particular kinds of facthood with particular kinds of distance—are *not* always the province of an unreflexive, reactionary cabal; that there *can* be neuroscientifically-wrought, biologically-correlated, methodologically-conservative factual imperatives, which seem to make some more—for want of a better word—“progressive” politics possible.

Perhaps our conclusion over-extrapolates from the data. But that is partly the point. Because this experiment not only moves us away from a negatively-biased social psychology more broadly—it legitimates and actualizes a whole series of other statements, both about human decision-making *and* about the virtues of collectivity in general. Indeed, this set of statements was subsequently expanded by experiments that pushed at the edges of this claim, showing how, in the scenario investigated by Bahrami et al. the collective benefit of the group emerges over time (Bahrami et al., 2012), and how this phenomenon is based on increased alignment in the language used to express confidence (Fusaroli et al., 2012). Moreover, if at a meta-theoretical level, the goal of the experiment was to reveal strengths in social interaction, at a methodological level the experiment was rigorously designed, and it precisely tested competing quantitative models. Thus, if it was critical of the way that social psychology had conjured the virtues of interaction, it made this critique entirely from within the confines of the social-psychological experimental game itself. Again, if it is only one case, the OIM study might be interpreted in terms of a broader, revised invocation of “the social” within social psychology; but it has also had an impact beyond these discussions –having been covered extensively by mainstream media outlets, and forming the basis for new experimental and meta-theoretical discourses about the virtues of information-sharing and open argumentation (e.g., Kanai and Banissy, 2010; Frith, 2012; Mercier and Sperber, 2012).

Before we concretize our analysis, let us add some important qualifiers to this story. Needless to say, first, this study might have gone another way; it might well have ended up re-enforcing the same conservative assumptions about the virtues of the private individual. But what’s interesting to us is that it didn’t: what captures our attention is the realization that such a conservative tendency is no special ally of the context-denying experimental game. Our core claim is that OIM is an example of a study which has not sought to examine its own biases and prejudices; nor has it attempted to disrupt the overt exclusion of political and economic contexts from experimental spaces; this is an experiment, by contrast, whose adherence to the *internal* rules of the experimental game of the neurosciences, whose delicate manipulation of the accepted and canonical parameters for these kinds of interventions, whose sensitivity to the dynamics of banality and strangeness within neuroscientific experimentation, has allowed it to enact, propel, and legitimate, what might otherwise be regarded as a progressive—even critical—intervention. But here we add a second caution: if our claim for the critical nature of this intervention relies on a shift in the psychological gaze from the capacities of the individual to the virtues of the collective, there is also a literature that locates the governing power and force of contemporary capitalism precisely in a shift to the efficiencies of flexible networks, and networked societies (Sennett, 1999; Castells, 2000; Boltanski and Chiapello, 2007). Through such a lens, OIM might be read as a demonstration—even a biologization—of the productive efficiency of networked collectivity (see especially Hartmann, 2012, on the relationships between neuroscience and network capitalism). In this paper, however, we do not claim that to move from the individual to the collective is necessarily “progressive” or “good”—experiments, Rheinberger (1994) reminds us, often have many stories to tell. *Our* story is bound up with the psychological elevation of the private individual (Danziger, 1994), an historical focus in that discipline on the negative effects of group interaction (Pepitone, 1981), and contemporary strategies for governance that *still* rely on the imagination of an atomized, psychologized, individual subject (see Slaby, 2010: 407, for a potent, neurobiologically-inflected example). It is in terms of this—still present—history that we want to interpret OIM as a critical intervention, and as a resource for (even an ally of) those who still see much to critique in such formations. Of course this alliance remains always *in potentia*; it is precisely the potentiality of OIM, and the forms of alliance that may be drawn with and through it, that we direct attention to here.

## Enacting the critical

We have argued that the intellectual force of Critical Neuroscience turns on a very specific relationship between “critique” and “neuroscience”. We suggested, further, that re-focusing on the specifically *experimental* spaces of the new brain sciences might help us to re-think this relationship. And we described one case where the parameters of an experimental game produced and legitimated some potent, critical statements form *within* a social neuropsychology. In this final section, we draw out four implications of that account.

### Critique

We suggested earlier that at least three factors distinguish Critical Neuroscience: (1) it insists on a deeply classical notion of “critique”, specifically as a self-consciously emancipatory, anti-commercial, and anti-capitalist socio-critique, rooted in the “historico-political” mission of the Frankfurt School (Slaby and Choudhury, 2012: 29); (2) it argues that socio-critique is something (currently; not necessarily) *exterior* to the experimental practices of the new brain sciences (if not to those sciences as such); and (3) it proposes further that the “brain fact” is but one node within a closed loop of mental phenomena, media representations, political and economic contexts, and so on, and that critical intervention is thus “*studying* the journey of a phenomenon in and around the neuroscience lab”—but it is never that journey itself (Choudhury et al., 2009: 64. Our emphasis).

We are much in sympathy with these arguments. But we use the OIM experiment to show that, even if we wish to hold onto such a notion of “critique”, we do not necessarily need the form of inquiry that these authors propose. The OIM experiment shows that, sometimes, nothing more than playing within the conventions and procedures that make up the experimental setting is required for the enactment of a prominent, and widely-publicized critique of—in this case—the deeply troubling, and intensely political, *individualizing* tendencies of much contemporary psychology and neuroscience. Unquestionably, the experiment, published in *Science*, created a very traditional, context-denying, unimpeachably “scientific” new “brain-fact”. And yet still this modest fact makes many strikingly critical statements possible—even necessary—within the new brain sciences: human beings often work sub-optimally when they work alone; collectivity trumps individualization, at least under some conditions; the communication of evidence is a delicate and subtle process; accurate knowledge is less an attribute of the single individual than a product of careful co-operation—so, on and on, go the claims that can be made. We argue that, irrespective of our notion of critique, we still need to extend our notion of a critical apparatus *beyond* an externally-focused, experimentally-indifferent, text-based form of (sometimes) scholastic nit-picking. Critical Neuroscience, if it is to further its contribution, might recognize that critique can be *in* brain-facts too.

### Politics

There is an odd correspondence between, on the one hand, some of the central assumptions of Critical Neuroscience and, on the other, general enthusiasm about the power and force of the contemporary neurosciences (e.g., Lynch and Laursen, 2010). If on first glance these seem like diametrically opposed literatures, still neither of them sees the neurobiological experiment, and the formal rules that demarcate it, as a way of potentially *doing* political critique (Fitzgerald and Callard, Forthcoming). Critical neuroscience is insistent that the formal laboratory-space of the contemporary neurosciences, and the regulations that govern it, are—rightly or wrongly; accurately or mistakenly—a more-or-less politics-free zone (Slaby, 2010: 406; Slaby and Choudhury, 2012: 39). By contrast, we have drawn on the OIM experiment to show that politics might sometimes be found in strange places (even occasionally outside of Frankfurt); that it can be expressed through some unexpected experimental practices (even in some quantitatively-specified neuroscientific modeling techniques).

Defenders of Critical Neuroscience might here say that we have missed the point; the whole purpose of these authors’ intervention has been to say that the seclusion from the political is merely rhetorical, that the experiment is of course always-already profoundly ideological (Slaby and Choudhury, 2012: 31). The main issue, moreover, is not a lack of politics, but the *wrong* politics—neuroscientific experiments are laid low by the “surprising parallel” between “cutting-edge neuroscience”, “organizational and management literature” and “neoliberal politics” (Slaby, 2010: 405). But the OIM experiment allows us to come at this relationship from quite a different angle—one that is intended to illuminate, rather than dispute, its characterization in the Critical Neuroscience literature. First, via its critique of the individualizing tendencies of social psychology, it shows us concretely how even the “depoliticalized” *rhetorical game* *of experimentation* might not be as demure as it first appears—that a carefully assembled, straightforward neuroscientific experiment can make a (potentially) critical intervention. So if we want to understand the relationship between politics and neuroscience, we will need some detailed account of experimental practices, and of the kinds of claims that those practices make possible. Second, it shows us how the politics expressed *through* this game can serve some “progressive” ends, such as provincializing the negative bias towards human sociality within contemporary psychology, and enabling a stream of experimentation on the potency and primacy of interaction and cooperation—a research-finding arguably quite inimical to at least some contemporary forms of neoliberal politics (but note again our qualifiers to this conclusion, discussed above). To realize this potency, you need to know the rules of the game—but you also need to know how those rules might be used for subtle and surprising ends. In order to understand the relationship between neuroscience and the potential for a more emancipatory, collective claim, you could do a lot worse than pay attention to classically-constituted psychophysical experiments.

### Context

Re-positioning experiments in their “context” is central to the mission of Critical Neuroscience: scientists should “be involved in the analysis of ‘contextual’ factors”, (Slaby, 2010: 397); “neuroscientists need to critically examine scientific practices and institutions as well as the wider social contexts within which they work” (Choudhury et al., 2009: 65); “the gathering of context in many cases may end up laying bare the economic and political imperatives that sustain particular styles of thought” (Slaby and Choudhury, 2012: 35). Over and over again, reversion to “context” is positioned as the weapon wielded by the critical imperative: it opens up the black-box of experimentation, allowing experimenters to see the ideological constraints within which they produce and disseminate knowledge. But we have tried to show that the relationship between the experiment and its context is not so straightforward—that the elision of context is not always a mark of deficit.

What makes experimental demonstration so potent, of course, is that the rules of the game require everyone to pretend that context doesn’t exist—that facts are somehow independent of the alarmingly human and social circumstances in which they have been assembled (Latour, 1987). We have no interest, here, in re-running philosophical or social-scientific debates about the structures of experiment (for which see e.g., Moghaddam and Harré, 1992; Shapin, 2010; Rheinberger, 2011). But we *are* interested in seeing how the formal elision of context (whether or not we agree with it; whether or not we think it’s ever actually enacted in practice) allows researchers to do and say particular kinds of things—and not all of them bad, or reactionary. What we have tried to show with the OIM demonstration is that the context-denying performance of an experimental game does *not* have to traduce the critical imperative—that it can, in fact, enact and legitimate a whole slew of critical interventions. More importantly, perhaps, to the extent that neuroscientific facts are (of course) always-already embedded in a context—then the interesting question might be less “how can we use ‘context’ to destabilize the facts that already exist?”; and more, “how can we play with context—including hiding it, denying it, excluding it—to facilitate the generation of facts that we want to bring into being *ourselves*?” (*cf*. Latour, 2010). It is an attention to experiment that is the radical gesture, here; not context. And it is precisely that quality of attention that allows these scholars to enact a *Science*-sanctioned sign of resistance to conventional thinking in the field (Fleck, 1979; *cf*. Roepstorff, 2002).

### Experiment

So how, finally, are we to think about neuroscientific experiments? More specifically: what should our attitude towards them be? How *seriously* should we take them? As we suggested earlier, an odd feature of much external commentary on neuroscientific experiment is its suspension between two poles: on the one hand, experiments are taken very seriously indeed. This is the experiment as a kind of reductionist terror, producing highly technified, publicly-valued, severely-reduced studies, which are “thought to be assuming the role of guidance in many people’s lives, both practically and through being incorporated into their self-understanding” (Choudhury et al., 2009: 63). On the other hand, experimental knowledge is not taken seriously at all. This is the experiment as thinly-veiled word-game, one whose thin, mediatized claims are easily taken apart once we “scrutinize and lay bare scientific conventions that are taken for granted, [as well as] tacit knowledge, [and] vested interests at work in neuroscience research or their impacts on people” (Slaby and Choudhury, 2012: 39). These are caricatures of complex arguments, of course—but they help us to draw attention to this strange back-and-forth between terror and dismissal.

By drawing attention to the OIM experiment, we argue for a Goldilocks approach within the sociology and philosophy of neuroscience: experiments should be taken *just seriously enough*. Throughout this paper, we have described the OIM experiment’s adherence to the “rules of a game”, and the care with which it arranges the props, tools, conventions, apparatuses and devices that make neuroscientific knowledge possible. But we also think that rules, games, tools, conventions and props are non-trivial things. To recognize this experiment as a clever move, within a specific game, is to both take it for what it is, and *value* it for what it is. We have shown how it was precisely an awareness of, and adherence to, the rigors and rules of the experimental game that allowed these scientists to intervene in unexpected, critical ways, and even to generate new “progressive” claims about collectivity and individuality. Indeed, we want to suggest that not only do experiments not have to be (either scientifically or politically) perfect to be interesting; it is precisely because they are *not* perfect that they *are* interesting (Rheinberger, 1994). The OIM experiment takes the experimental game just seriously enough to have its claims treated as consequential and impactful; but not so seriously that it cannot play within the taken-for-granted rules of the game, that it cannot *thereby* reach out to broader political and social landscapes, that it cannot produce other kinds of strange statements too. Not for nothing does the paper conclude its sober analysis about success in locating good evidence *between* individuals with the observation that “we know all too well about the catastrophic consequences of consulting ‘evidence’ of unknown reliability on problems as diverse as the existence of weapons of mass destruction and the possibility of risk-free investments” (Bahrami et al., 2010: 1084). Being able to make such a claim, in a journal like *Science*, is precisely what we refer to when we urge commentators to learn to take experiments “just seriously enough”.

## Conclusion

So what *should* be the Critical in Critical Neuroscience? After all, much as the Critical Neuroscientists look upon neuroscience itself, we are not invested in tearing down Critical Neuroscience—merely inviting it to reflect on the biases and assumptions inherent in its own approach, asking it to examine the forms of politics it enacts, and encouraging it, in its turn, to consider especially the methodological assumptions through which it enacts them. We have tried to show that, in the rush to critique and to reform neuroscience; in the desire to remake it as a more socially and politically incisive discipline; in the stern denunciation of its ties to managerial, pharmaceutical and other spaces of neoliberal accumulation; in the demand that it be reflexive, and self-critical, and more obviously and openly aware of its own surroundings—in all of this, broader questions about how critique might be enacted *within* the mundane rules of the contemporary neuroscientific game have been missed. We have argued that, *contra* the literature up to now, the first question for a specifically *critical* neuroscience needs to be: what can neuroscience do? We do not here offer any kind of comprehensive answer. But what we have tried to gesture at is the realization that if neuroscience can help to govern and surveil us; if it can pathologize us and reduce us to our biological parts; if it can induce us to buy drugs, nudge us to change our behavior, and help us to become more rigidly bourgeois in our parenting—then surely it can also help us to imagine and enact ourselves, and our societies, and the political, economic and cultural assumptions on which those societies are organized, in some more interesting and hopeful ways too. We have demonstrated just one minor instance of how this might work, and how a critical political statement can be made possible entirely within the constraints of contemporary experimental practice. But our central contribution has been to argue that the critical imaginary of Critical Neuroscience, if it does not require radical reform, at least needs a broader sense of its own possibility. We have urged attention to the critical impetus of experiment, here—but there are likely many similar arguments waiting to be made.

At the end of their 2009 programmatic contribution, Choudhury et al. point out that the neurosciences are making progress further and further into areas that were once dominated by the humanities, the social sciences, the clinical arts, and so on (73–74). But their own goal, they stress, is less keeping the neurosciences out of these domains, but in creating the ground for “critical engagement” that will ultimately “drive new ideas for experiments in neurosciences” (ibid.). They wish to show sociologists and anthropologists how they, too, might help “to influence the shape of future research in neuroscience” (ibid.). Here, we are in total agreement with Choudhury et al. But our suggestion is that such engagement is unlikely to come from an instance on implacable context; indeed, the central gambit of this paper is that it might be more “experiment”, and less “context”, that forms the meeting-ground between neuro-biological and socio-political research practices in the 21st century. We join with our colleagues in philosophy and the social sciences, and even in critical theory, who are interested in the practices and effects of the neuroscientific laboratory. But what we want to stress is that, if they do cross the experimental threshold, it might not be so unthinkable for such scholars to just run with the rules of the game as they find them. In other words, it might not be so terrible to go into the experiment, and to leave the world where it is for a moment; to set aside an otherwise valuable attention to “politics”, and “interest”; to leave the workings of “society” and “culture” where they are; and finally, even if only temporarily, to (quietly) close the laboratory door.

## Conflict of interest statement

The authors declare that the research was conducted in the absence of any commercial or financial relationships that could be construed as a potential conflict of interest.
